# Reassessment of the importance of mucins in determining sputum properties in cystic fibrosis

**DOI:** 10.1016/j.jcf.2013.11.002

**Published:** 2014-05

**Authors:** Alex Horsley, Karine Rousseau, Caroline Ridley, William Flight, Andrew Jones, Thomas A. Waigh, David J. Thornton

**Affiliations:** a Manchester Adult Cystic Fibrosis Centre, University Hospital South Manchester, Manchester M23 9LT, UK; b Institute of Inflammation and Repair, Education and Research Centre, University Hospital South Manchester, Manchester M23 9LT, UK; c Wellcome Trust Centre for Cell-Matrix Research, Faculty of Life Sciences, University of Manchester, Manchester M13 9PT, UK; d Biological Physics, School of Physics and Astronomy, University of Manchester, Manchester M13 9PL, UK

**Keywords:** Cystic fibrosis, Mucin, Proteolysis, Lung inflammation

## Abstract

**Background:**

There is conflicting evidence about the importance of airway mucins (MUC5AC and MUC5B) in determining physical properties of sputum in cystic fibrosis (CF). We studied the effects of endogenous degradation of mucins on CF sputum elasticity and apparent mucin concentrations.

**Methods:**

Elastic shear moduli (G′) and mucin concentrations in sputum of 12 CF patients were measured before and after incubation at 37 °C for 60 min.

**Results:**

G′ fell from a median of 5.98 to 4.70 Pa (p = 0.01). There were significant falls in MUC5AC (8.2 to 5.2 μg/ml, p = 0.02) and MUC5B (17.3 to 12.5 μg/ml, p = 0.02) over the same period, and associated decrease in molecular weight and size.

**Conclusions:**

Sputum is not inert and degradation reduces apparent mucin concentrations and sputum elasticity. Even if care is taken to process samples rapidly, sputum may therefore differ from secretions retained in airways. Previous studies may have underestimated the role of mucins in CF sputum.

## 1 Introduction

Cystic fibrosis (CF) is characterized by chronic airway infection and inflammation, and clinically by accumulation of airway secretions [1]. Clearance of these secretions is a major objective of CF care, typically involving daily physiotherapy [2]. The physical properties of sputum, which determines the ease or otherwise of airway clearance strategies, reflect a complex interaction between different macromolecular components, ions and hydration. The influence of these different components however is incompletely understood.

Sputum is a complex mix of airway mucins, DNA, proteins, and bacterial and host inflammatory cells [3], [4]. This is quite distinct from normal airway mucus which consists predominantly of water, ions, proteins and the secreted airway mucins MUC5AC and MUC5B [5]. In the healthy airway, these polydisperse large molecular weight glycoproteins perform an important role in airway defence and hydration [5]. MUC5AC is secreted mainly from goblet cells in the surface respiratory epithelium and MUC5B predominantly from submucosal glands [6].

Mucin production is upregulated in response to a range of airway insults [7]. In CF, the airway is exposed to a wide range of such stimuli, including endobronchial infection with *Pseudomonas aeruginosa* [8] and *Staphylococcus aureus* [9], that have been shown to activate NF-κB and upregulate mucin gene transcription [7]. Both goblet cell hyperplasia [6] and increased submucosal gland volume [10] have been described in CF airway biopsies, suggesting either a chronic increase in production of mucins or a failure to release mucins from goblet cells [11].

Despite the central role of airway secretions in the pathophysiology and symptomatology of CF, there is disagreement about the importance of mucins in determining the physical properties of CF sputum. Reliably identifying different mucin subtypes is a challenging and complex process involving solubilization and separation of mucins, and recognition by specific antibodies [12]. Early analyses indicated that, weight for weight, there was around twice as much mucin as DNA in CF sputum [13]. More recent attempts at quantification of mucin levels in CF however have shown intriguing results, with levels of both MUC5AC and MUC5B reportedly substantially lower in CF sputum than in secretions from healthy airways [11]. Although subsequently shown to increase during a pulmonary exacerbation, mucin levels remained the same or less than those found in healthy secretions [14]. This raises questions about the importance of mucins in CF sputum. Could low mucin levels be a consequence of bacterial infection (e.g. due to mucin degradation) or an underlying cause (e.g. might altered mucin profiles be responsible for the characteristic infections seen in CF)? Airway blockage seen in very young children with no evidence of infection [15] suggests that in the absence of large amounts of bacterial and inflammatory cell DNA mucins alone may be responsible for airway obstruction. Greater understanding of the role of mucins in airway pathophysiology is important therefore at all stages of this complex disease, and has implications for future research and treatments.

We hypothesized that mucins were subject to significant degradation within sputum from CF patients, and that this could explain the previous conflicting evidence on the role of mucins in CF sputum. We assessed mucin degradation in vitro by examining the effect of incubation at 37 °C on sputum elasticity and airway mucin concentrations. The aims of this study were thus to: 1) assess the effect of endogenous degradation on elasticity of CF sputum; 2) assess the effect of depolymerization of DNA and mucins on elasticity; 3) quantify the levels of airway mucins MUC5AC and MUC5B in CF sputum; and 4) assess the effects of degradation of MUC5AC and MUC5B on apparent mucin concentrations.

## 2 Methods

### 2.1 Subjects

This was an observational study of adults with CF undergoing treatment for a pulmonary exacerbation. Exacerbation was defined clinically and all patients were assessed within 24 h of commencement of therapy.

Spirometry was performed according to ATS/ERS guidelines. Baseline forced expiratory volume in 1 s (FEV_1_) was taken as the highest in the preceding 6 months. Body mass index (BMI) was calculated at the time of sputum collection. 24 h sputum load was given as the total weight of sputum expectorated over the preceding 24 h.

The study was approved by the North West Research Ethics Committee (study reference 10/H1014/71) and all patients provided written informed consent.

### 2.2 Sputum processing

Sputum samples were collected by spontaneous expectoration over no more than 5 min and transported on ice for rapid processing (collection to storage within 30 min). Whole sputum was transferred to a sterile Petri dish and sputum samples separated out into pre-weighed tubes. Samples were frozen at − 80 °C for storage prior to analysis.

### 2.3 Elasticity

Following thawing at 37 °C for 5 min, 100 μl of sputum was aliquoted using a positive displacement pipette (Brand GMBH, Wertheim, Germany). Rheology was assessed using a controlled stress rheometer (Bohlin HR Gemini, Malvern Instruments, Worcestershire, UK) and 20 mm/2° cone maintained at 37 °C by the Peltier plate. Sample drying was prevented by application of a thin layer of mineral oil (Sigma-Aldrich, Dorset, UK) around the edge of the cone. Storage (elastic) shear moduli (G′), reflecting solid-like properties or overall degree of cross-linking in the sample, and loss (viscous) shear moduli (G″) were calculated from the measured response of the samples to an oscillating angular displacement. Frequency sweeps were performed between 0.5–10 Hz with a stress of 1 Pa (within the linear viscoelastic range). Data are quoted as the mean of 3 repeats at 1 Hz.

### 2.4 Degradation of mucins and DNA

In order to assess the effect of endogenous degradative processes, serial rheological and mucin analyses were made at intervals after commencing incubation at 37 °C. Sputum was gently mixed prior to removal of aliquots. All samples were assayed at time 0 and 60 min.

Two samples with minimal rheological change on simple incubation were subjected to additional analyses to assess the relative contribution of DNA and mucins to overall sputum elasticity. Each of three 200 μl aliquots was treated with 10 μl of either 0.9% saline, recombinant human DNase (final concentration 2.5 μg/ml) or dithiothreitol (DTT) (final concentration of 10 mM). Half was analyzed immediately and the remainder was incubated at 37 °C for 60 min before elasticity measurements were repeated.

### 2.5 Quantification of MUC5AC and MUC5B

Mucins were separated by agarose gel electrophoresis and Western blotting, as previously described [12]. Mucins were reduced prior to separation by addition of 10 μl 100 mM DTT and heating to 100 °C for 15 min, or separated whole and reduced prior to transfer by soaking the gel in 200 ml 0.6 M NaCl/60 mM sodium citrate buffer (SSC) containing 10 mM DTT for 30 min. The polyclonal antibodies MAN5AC-I and MAN5BIII were used to identify separated mucins, MUC5AC and MUC5B respectively [16], and concentrations were derived from a standard curve of purified mucins, run on the same gel [17], [18].

### 2.6 Mucin purification & molecular weight determination

Selected samples were purified using a two-step isopycnic density gradient centrifugation procedure, as previously described [19]. The weight-average molecular weight (Mw) and the weight-average mean square radius (Rw) of mucins were determined by size-exclusion chromatography and multi-angle laser light scattering (SEC-MALLS), as described previously [17].

### 2.7 Statistical analysis

Data were analyzed using Prism (GraphPad Software Inc., CA, USA). Normal distribution was assessed using the D'Agostino and Pearson omnibus normality test. Results are quoted as mean (SD) unless otherwise stated. Skewed data were log-transformed prior to analysis. Paired t-test was used for comparison of change in variables between paired time points. A p value of below 0.05 was considered as statistically significant.

## 3 Results

Data are available on 12 subjects (Table 1), median age of 22 years. All patients were chronically infected with *P. aeruginosa*. The majority of patients had significant lung function impairment with a median baseline FEV_1_ of 37.8% predicted.

**Table 1:** Demographic and clinical data.

Male/female	7/5
Age (years)	22 (18–43)
Best FEV_1_% predicted in previous 6 months	37.8 (28.6–90.8)
FEV_1_% predicted at start of exacerbation	30.8 (19.1–44.4)⁎
BMI (kg/m^2^)	20.4 (17.1–22.3)
24 h sputum weight (g)	23 (12–129)

Data are presented as median (range). FEV_1_: forced expiratory volume in 1 s. BMI: body mass index.

⁎ p = 0.0005 compared to baseline.

### 3.1 Sputum rheology

**Fig. 1:**
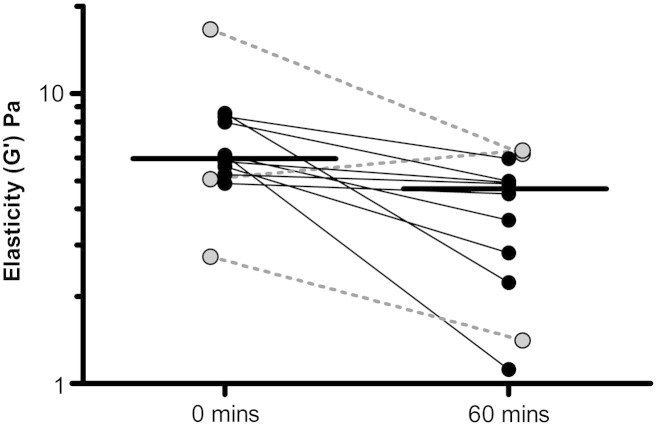
Change in elastic modulus (G′) of CF sputum samples incubated at 37 °C for 60 min. Each pair of points represents the same sputum sample assayed at 2 time points, horizontal bars represent group medians. 3 patients who had received nebulized DNase within 12 h of sampling are offset and highlighted in gray with dotted joining bars. There was no difference in baseline elastic modulus between these three and the remaining 9 patients, nor in G′ change with incubation.

Sputum samples from 12 patients underwent paired rheological analysis before and after incubation at 37 °C for 60 min. Median (range) baseline elastic modulus (G′) was 5.98 (2.74–16.6) Pa. This fell significantly to 4.70 (1.12–6.36) Pa at 60 min (p = 0.01), see Fig. 1.

In two larger volume samples with minimal G′ change on incubation, DNase and DTT each caused larger falls in elastic modulus than incubation with saline. Mucin depolymerization by DTT appeared to have greatest effect on sputum elasticity: mean change in G′ with saline was + 19% (2.80 to 3.36 Pa), mean change with DNase was − 25% (2.62 to 1.77 Pa) and mean change with DTT was − 54% (2.16 to 0.80 Pa).

### 3.2 MUC5AC concentration

Paired MUC5AC analyses were available on 9 patients: in two cases there was insufficient sample and one case was excluded at 60 min because of processing error.

**Fig. 2:**
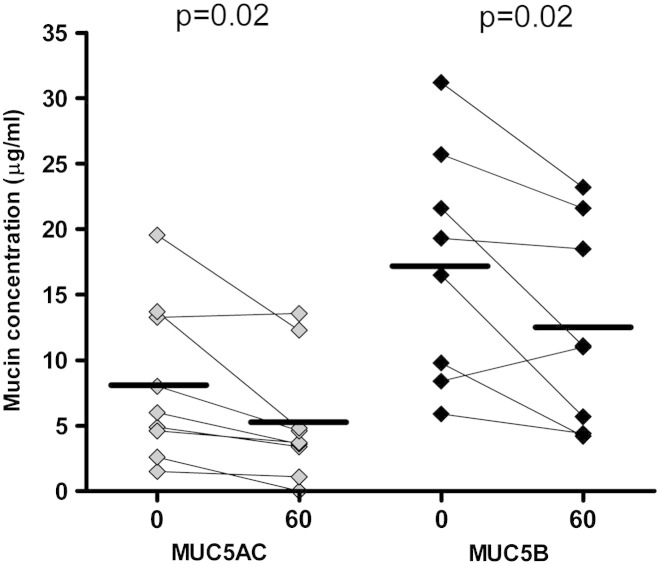
Change in MUC5AC and MUC5B immuno-reactivity with incubation at 37 °C. Each pair of points represents sampling of the same sputum sample at different time points. Horizontal bars represent group means.

**Fig. 3:**
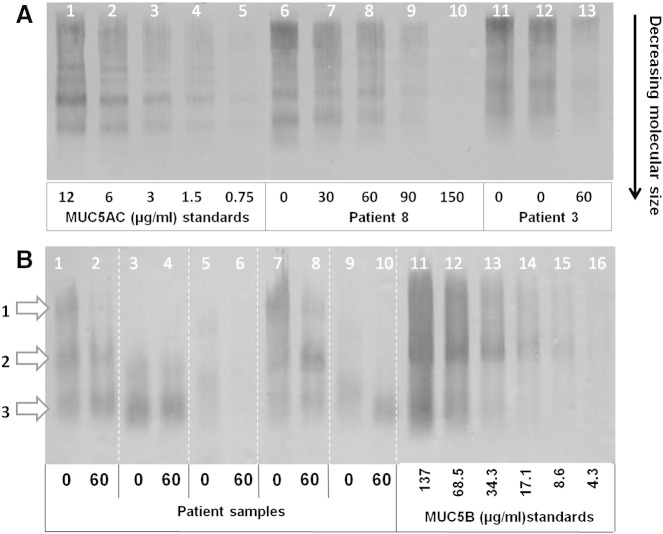
Representative section of Western blot of sputum samples showing (A) MUC5AC and (B) MUC5B immunoreactivity. Serial doubling dilutions of mucin standards (dilution given below band) are given for each mucin. 3A: Lanes 6–10 represent a single sputum sample from one patient, sampled at multiple time points during incubation at 37 °C (time given in minutes below the bands). Lanes 11–13 represent analyses of a sputum sample from a second patient during incubation at 37 °C, including 2 repeats from time 0 (sample times given in minutes). Different bands represent different molecular weight or polymeric forms of MUC5AC [17]. Bands appearing at the top of the gel represent the most highly polymerized forms, whose large molecular size retards progression through the gel. 3B: Vertical white lines separate samples from different subjects. White arrows on the left indicate the different forms of MUC5B; 1 — low charge glycoform, 2 — high charge glycoform, 3 — degraded mucin fragments [20].

There was a significant fall in mean (SD) MUC5AC immunoreactivity after incubation, from 8.2 (6.0) to 5.2 (4.7) μg/ml (37% reduction, p = 0.02), see Fig. 2. Mucins were not reduced prior to electrophoresis, allowing visualization of different sized mucin polymers (larger polymeric forms migrate less rapidly [17]). A representative section of a Western blot is shown in Fig. 3A. Although signal intensity decreased with time, there did not appear to be a change in the configuration of mucin bands, consistent with loss of immunoreactive epitope rather than depolymerization to smaller forms.

### 3.3 MUC5B concentration

Paired MUC5B data are available from 8 patients (insufficient sputum in one additional case). Mean (SD) MUC5B concentration was greater than MUC5AC: 17.3 (8.9) μg/ml vs 8.5 (6.4) μg/ml (p = 0.009). Mean (SD) MUC5B fell from 17.3 (8.9) μg/ml at time 0 to 12.5 (7.7) μg/ml after incubation (28% reduction, p = 0.02) (see Fig. 2). To permit separation in gels, MUC5B samples required reduction and appear in three bands, representing 2 different glycoforms (upper 2 bands) and a lower band of degraded mucins [20] (see Fig. 3B). Unlike MUC5AC, there was not only a reduction in signal intensity but also a change in configuration of the bands (i.e. relative disappearance of less mobile bands).

### 3.4 Mucin molecular weight and size

**Fig. 4:**
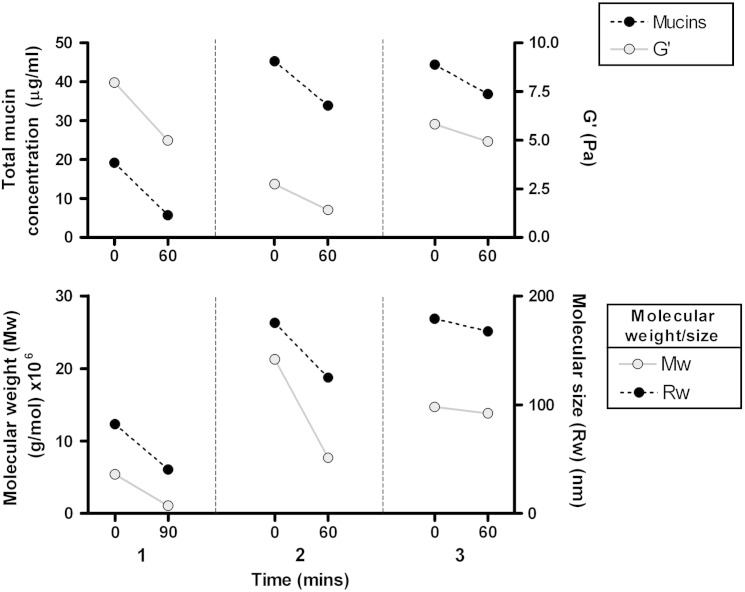
Change in total mucin concentration (black, dotted line) and elastic shear modulus (G′, gray) (top), and molecular weight (Mw, gray) and size (Rw, black, dotted line) of purified mucins from sputum of three different CF patients. Each vertical column represents paired data from separate patients (numbered 1–3) assessed before and after incubation at 37 °C for 60mins. Mw and Rw are shown at 90 min for sample 1 because of insufficient sample at 30 and 60 min for reliable quantification, though the appearances were the same.

Purified mucin fractions from the sputum of three patients were analyzed for molecular size (Rw) and weight (Mw) by SEC-MALLS before and after incubation. Changes in Mw and Rw in individual samples broadly mirrored those seen in mucin immuno-reactivity and rheology (see Fig. 4). Mucins in sample 1 appeared to be already substantially degraded at time 0, with lower Mw and Rw than the other 2 samples. As with the mucin concentrations in this sample, Mw and Rw both fell further after incubation.

## 4 Discussion

Understanding the nature of airway secretions is vital to understanding how CF progresses and, particularly in later stages of the disease, to developing therapies to assist airway clearance. This study provides additional findings that may help to explain some of the earlier confounding observations on the importance of mucins in CF sputum.

We have shown that both elasticity and mucin composition of sputum can be expected to decline rapidly at body temperature. In some subjects mucins within sputum appeared already substantially degraded at the start of the experiment. These data are consistent with previous findings that have shown a larger proportion of smaller polymeric mucins in CF sputum compared to the mucins in normal tracheobronchial secretions [13], [21], [22]. As previously described, MUC5B appeared to be the predominant mucin in CF sputum [12], though this may also reflect more rapid degradation of MUC5AC [21]. Although it has been recognized for some time that bacterial or host inflammatory cell proteases in the sputum may degrade mucins [23], only recently has the importance of such processes begun to be appreciated [24]. As with previous studies [12], [13], we also observed a wide range of mucin levels in CF sputum and we have gone on to show that this might represent differences in mucin degradation. This has important implications for our understanding of earlier observations. In particular, this may explain the finding that mucin levels are lower in CF sputum than healthy controls [11]. The time course of mucin degradation is rapid and highly variable, with an average 30% reduction in mucin levels at 1 h. Since it is probable that sputum has been residing within the proximal airways for some time before expectoration [25], and since it is also unlikely that mucins would depolymerize ex vivo but not in the airway, it is reasonable to anticipate that mucins may already have been subject to substantial degradation even in fresh sputum. Lower airway sputum plugs may therefore have quite different MUC5AC and MUC5B forms and apparent concentrations, and we cannot infer these from expectorated sputum. Retention of distal secretions may mean that they are subject to greater degrees of degradation. Alternatively, the mucins in dense plugs may be protected to a certain extent from the high levels of proteases found in the proximal airways. This is consistent with the observation by Burgel et al. that the lumen of small airways in CF lungs removed for transplantation were characterized by mucus plugs and obstruction [26].

We collected sputum samples from a single episode of expectoration, stored these on ice within 5 min and processed them rapidly. This contrasts with sputum in earlier studies which was collected over 30 min [11], [14]. In both studies, patients were chronically infected with *P. aeruginosa*, and a recent study has described the in vitro effect of *Pseudomonas*-derived proteases on mucin levels [24]. Although it is not possible to be certain whether the low mucin levels in CF sputum represent differences in mucin secretion or in their degradation by airway proteases, it is clear from our data that this latter process has an important influence on mucin immunoreactivity. Since patients in this study were undergoing an exacerbation, the role of this process in stable CF patients and in healthy subjects also requires future clarification.

We observed that as MUC5B degraded with time, there was a change in electrophoretic bands, indicating pututative differences in degradation of the different glycoforms. For MUC5AC however the signal appeared to decrease more uniformly and banding appearance was unchanged. This suggests a loss of epitopes for the MAN5AC-I antiserum rather than the breakdown of MUC5AC into smaller units, and the polymer may still be intact due to disulphide bonding. For this reason we also applied SEC-MALLS to selected large-volume mucin samples. This confirmed that the time-dependent fall in immuno-reactive signal was mirrored by changes in average molecular weight and size, consistent with depolymerization of the mucin chains. If degradation affected the two mucin forms differently however, with depolymerization occurring predominantly within the MUC5B fraction, long chains of MUC5AC might still contribute to the 3D gel structure of the sputum.

In this study we have not included secretions from healthy controls. These have been looked at previously and noted to show temperature dependent degradation over 24 h similar in nature to that observed here over 1 h, although in the earlier study other timepoints were not reported [27]. Although the focus of the current work was what was happening in CF, future studies to investigate this phenomenon would benefit from comparison with healthy controls and other disease groups.

Henke et al. concluded that the lower levels of mucins in CF, and the high concentrations of DNA, indicated a more important role for the latter in determining the physical characteristics of sputum [11], [28]. It is undoubtedly the case that DNA has an major influence on CF rheology [29], and this is borne out by the clinical impact of DNase and its role in airway clearance therapies. Our data however also support a role for mucins. In two samples that showed little or no change in rheology with incubation at 37 °C, greater impact on sputum elasticity was seen with DTT (a compound known to solubilize mucins by reducing disulphide bonds between mucin chains) than DNase. Although the numbers are small, these preliminary observations agree with earlier in vitro data on the impact of N-acetylcysteine on sputum rheology [30]. Trials of both nebulized and oral N-acetylcysteine however have been disappointing [31], and it is clear that a beneficial effect of mucin solubilization is not universal. CF is by nature a highly heterogeneous condition, with considerable differences in disease expression both between and within patients. The suggestion that inspissation of secretions is down to DNA alone has been based on sputum from patients with significant lung disease, and all with chronic infection with *P. aeruginosa*. The picture is further complicated by the binding of proteases to DNA, such that treatment with DNase may also permit greater degradation of mucins [32]. Even if DNA was the predominant polymer in freshly expectorated sputum, as emphasized here it would not necessarily hold true for secretions retained in the lungs of these patients. More importantly, it cannot be generalized to milder CF patients where the infective load is less and mucin levels may be higher [24]. Indeed, studies in early CF have detected physiological evidence of airway obstruction even in the absence of overt infection or inflammation [15]. Effective mucolytic therapies may therefore be important in some patients or at earlier stages of the disease.

In conclusion, we have provided important supporting evidence that degradation of mucins occurs in the sputum of CF patients with chronic infections. This occurs rapidly in both of the major secreted mucin subtypes. Expectorated sputum may therefore be quite different in elasticity and mucin composition from that sequestered more distally in the lung. At present we lack good tools to understand the nature of airway secretions and airway blockage in the small airways in vivo. Future work should address the role of mucins in the airways of patients free of *P. aeruginosa*. Important information could be obtained from lower airway samples, either obtained by hypertonic saline sputum induction or lower airway brushings. Therapies aimed at improving mucin solubilization may yet have an important role in airway clearance.
